# Development of Frataxin Gene Expression Measures for the Evaluation of Experimental Treatments in Friedreich’s Ataxia

**DOI:** 10.1371/journal.pone.0063958

**Published:** 2013-05-17

**Authors:** Heather L. Plasterer, Eric C. Deutsch, Matthew Belmonte, Elizabeth Egan, David R. Lynch, James R. Rusche

**Affiliations:** 1 Repligen Corporation, Waltham, Massachusetts, United States of America; 2 Departments of Neurology and Pediatrics, Perelman School of Medicine at the University of Pennsylvania, Philadelphia, Pennsylvania, United States of America; 3 Divisions of Neurology and Pediatrics, Children’s Hospital of Philadelphia, Philadelphia, Pennsylvania, United States of America; Thomas Jefferson University, United States of America

## Abstract

**Background:**

Friedreich ataxia is a progressive neurodegenerative disorder caused by GAA triplet repeat expansions or point mutations in the *FXN* gene and, ultimately, a deficiency in the levels of functional frataxin protein. Heterozygous carriers of the expansion express approximately 50% of normal frataxin levels yet manifest no clinical symptoms, suggesting that therapeutic approaches that increase frataxin may be effective even if frataxin is raised only to carrier levels. Small molecule HDAC inhibitor compounds increase frataxin mRNA and protein levels, and have beneficial effects in animal models of FRDA.

**Methodology/Principal Findings:**

To gather data supporting the use of frataxin as a therapeutic biomarker of drug response we characterized the intra-individual stability of frataxin over time, determined the contribution of frataxin from different components of blood, compared frataxin measures in different cell compartments, and demonstrated that frataxin increases are achieved in peripheral blood mononuclear cells. Frataxin mRNA and protein levels were stable with repeated sampling over four and 15 weeks. In the 15-week study, the average CV was 15.6% for protein and 18% for mRNA. Highest levels of frataxin in blood were in erythrocytes. As erythrocytes are not useful for frataxin assessment in many clinical trial situations, we confirmed that PBMCs and buccal swabs have frataxin levels equivalent to those of whole blood. In addition, a dose-dependent increase in frataxin was observed when PBMCs isolated from patient blood were treated with HDACi. Finally, higher frataxin levels predicted less severe neurological dysfunction and were associated with slower rates of neurological change.

**Conclusions/Significance:**

Our data support the use of frataxin as a biomarker of drug effect. Frataxin levels are stable over time and as such a 1.5 to 2-fold change would be detectable over normal biological fluctuations. Additionally, our data support buccal cells or PBMCs as sources for measuring frataxin protein in therapeutic trials.

## Introduction

Friedreich ataxia (FRDA) is an autosomal recessive neurodegenerative disorder caused by a GAA triplet repeat expansion or point mutations in the *FXN* gene. With a prevalence of 1 in 50,000 FRDA is the most common inherited ataxia. Most individuals with FRDA (97%) have an expanded GAA triplet repeat in the first intron of the frataxin gene in both alleles [1]. The triplet repeat leads to decreased frataxin mRNA transcription and, ultimately, a deficiency in the level of frataxin protein. The majority of the remaining 3% of patients have the triplet repeat expansion in one allele and a point mutation in the other allele [2]; such point mutations also lead to deficiency of functional frataxin.

Frataxin is a ubiquitous, nuclear-encoded mitochondrial protein [3]. The deficiency in frataxin is associated with iron accumulation in the mitochondria, impairment of Fe-S cluster containing enzymes, increased sensitivity to oxidative stress, and deficits of respiratory chain complex activities [4]–[6]. While frataxin is expressed in all tissues, FRDA mostly impacts neural and cardiac tissue for reasons that are not completely understood [7]–[9]. Clinical manifestations of the disease include ataxia, loss of coordination and reflexes, cardiomyopathy, scoliosis, diabetes and optic atrophy [10]–[12]. The age of onset of disease manifestations is variable, but typically occurs in late childhood or early adolescence and correlates with GAA repeat length on the shorter allele. There is no approved treatment for FRDA.

Individuals who are heterozygous “carriers” of the triplet GAA expansion have levels of frataxin that are approximately 50% of normal levels yet they manifest no clinical symptoms. This suggests that therapeutic approaches that increase frataxin may be effective even if frataxin is raised only to the level of a carrier. Small molecule HDAC inhibitor (HDACi) compounds increase histone acetylation at the frataxin locus and increase frataxin mRNA and protein levels [13]. Increases in frataxin mRNA and protein after HDACi treatment have been observed *in vitro* using primary lymphocytes isolated from patient blood [14]. Beneficial effects of HDACi have also been documented in animal models of FRDA [14]–[16].

The interpretation of clinical trials in FRDA with agents that change frataxin expression will require biological markers of drug response. A variety of clinical measures including examination-based scales and performance measure composites have been validated in FRDA, but their sensitivity to change as reflected in natural history data is limited. Similarly, biochemical markers of FRDA are at present limited. Techniques to measure peripheral frataxin levels have been developed, but many of the practical features of their use (such as short-term and long-term variability independent of therapy) have not yet been defined [17]–[20]. In addition, data linking serial clinical change with frataxin level, confirming frataxin levels as appropriate targets in the disease, have not yet been obtained.

Here we characterize the correlation of frataxin protein levels with disease severity, the stability of frataxin levels over time, and the contribution of frataxin from different components of whole blood. We then demonstrate that increases in frataxin mRNA and protein can be quantified in peripheral blood mononuclear cells (PBMCs) following HDACi treatment. Finally, we investigate predictive ability of frataxin measurements on neurological progression, and discuss its impact as a biomarker in measuring response to therapies aimed at increasing frataxin.

## Methods

### Ethics Statement

All protocols were approved by the IRB at the University of Pennsylvania or the Children’s Hospital of Philadelphia, or by the Ethics Committee at Erasme Hospital, Brussels, Belgium, and written informed consent was obtained from each subject.

### Patients

Cohort 1 contained individuals enrolled in a clinical trial of an agent unrelated to frataxin expression [21] while cohort 3 were randomly selected subjects from the clinical populations of the Children’s Hospital of Philadelphia. Clinical and demographic information for patients (Cohort 3) involved in *ex vivo* studies (GAA repeat length, age, etc.) were typically self-reported or derived from the Friedreich Ataxia Clinical Outcome Measures database (n = 98) [22]. Cohort 2 contained individuals selected based on the ability of a patient and related carrier to provide weekly blood donations for a 15-week duration. Clinical information was not collected from cohort 2 subjects.

### Whole Blood Collection and Fractionation

Blood was drawn into K_2_EDTA Vacutainer tubes (BD Biosciences, San Jose, CA) unless specified otherwise and shipped overnight at ambient temperature. Upon receipt, blood was either frozen at −80°C until further processing or was immediately processed for isolation of erythrocytes or PBMCs.

For fractionation of whole blood, freshly drawn blood was separated into fractions that were enriched for plasma, PBMCs, platelets, granulocytes, or erythrocytes. First, 4.5 mL of whole blood was diluted 1∶2 in phosphate buffered saline (PBS) and slowly layered over 6 mL lymphocyte separation medium (LSM). The blood was centrifuged for 40 minutes at 1,000 rpm at room temperature. After separation, the top plasma layer, the “buffy coat” layer containing PBMC, and the LSM layer were transferred to new 15 mL conical tubes. The volume of the tubes containing plasma, LSM, and the remaining erythrocyte pellet were each increased to 9 mL with PBS. The volume of the tube containing PBMCs was increased to 4.5 mL with PBS. To isolate platelets, the PBMC fraction was centrifuged an additional two times for 10 minutes at 1,000 rpm at room temperature. After the first spin, the supernatant containing platelets was transferred into a new 15 mL conical tube, the PBMC pellet was resuspended in 4.5 mL PBS, centrifuged, and the supernatant was transferred into the conical tube containing platelets. The PBMC pellet was then resuspended in 9 mL PBS.

In a parallel, but separate fractionation procedure, 4.5 mL of whole blood was diluted 1∶2 in 1×PBS, centrifuged, and the erythrocyte pellet was further separated into granulocytes using three, sequential washes with 10 mL Erythrocyte Lysis Buffer (Buffer EL) (Qiagen, Valencia, CA). Each wash with Buffer EL was performed for 5 minutes at room temperature and was followed by centrifugation at 1,000 rpm for 10 minutes at room temperature. After the third wash, the granulocyte pellet was resuspended in 9 mL in PBS and stored at −80°C until further processing or was immediately processed for frataxin protein measurements.

PBMCs were isolated from approximately 30 mL of EDTA-anticoagulated whole blood using differential centrifugation and Lymphocyte Separation Medium (LSM) (MP Biomedicals, Solon, OH) or, alternatively, by transferring the blood into Cell Preparation Tubes (BD Biosciences). After separation, PBMCs were washed with PBS, incubated for 5–10 minutes with Buffer EL to ensure complete removal of erythrocytes, and then washed a final time with PBS to remove residual Buffer EL. Cell pellets were then stored at −80°C until further processing or resuspended in cell culture medium and treated with HDACi as described below.

### Treatment of PBMCs with HDAC Inhibitors

Freshly isolated PBMCs were cultured in RPMI 1640 medium supplemented with 15% FBS, L-glutamine, penicillin, and streptomycin. Cells were cultured in 6-well plates in the presence of the HDAC inhibitor RGFP109 or DMSO as a vehicle control. RGFP109 was added to cells for a final concentration of 1, 2.5, 5, or 10 µM, and DMSO was added for a final 0.1% concentration. Cells were incubated for 48 or 72 hours before harvesting total mRNA or protein. Total mRNA was isolated using an RNeasy Mini Kit (Qiagen) following the manufacturer’s protocol and including an optional on-column DNAse digestion.

### Buccal Cell Collection

Buccal cells were collected for frataxin protein analysis as previously described A total of three swabs were used for each subject (alternating cheeks with each swab), which were combined in a single microcentrifuge tube containing 500 µL of ice-cold Extraction Buffer A (Abcam, Cambridge, MA). After the final swab was collected, the tube was immediately placed on dry ice and stored at −80°C until further processing. To extract protein from the buccal cells, the microcentrifuge tubes were thawed on ice, incubated on ice for 20 minutes with intermittent vortexing, and centrifuged at 20,000 g for 10 min at 4°C. The cell supernatant containing soluble protein was transferred to a new tube and protein was quantified using a BCA protein assay according to the manufacturer’s protocol (Pierce Protein Research Products, Thermo Fisher Scientific, Rockford, IL).

### Frataxin Protein Measures

Frataxin protein was measured using either a dipstick lateral flow immunoassay (Abcam) or an ELISA (Abcam). To compare the interchangeability of the two frataxin assay formats, we measured frataxin protein levels in PBMC extracts from five individuals in triplicate using both methods, and the amount of frataxin was quantified based on a rFXN standard curve. The levels of frataxin as determined by each method were highly correlated (R^2^ = 0.845).

The lateral flow immunoassay (Abcam) was performed as previously described [23]. Briefly, 25 µg of buccal cell protein in 25 µL of Buffer A was mixed with 25 µL blocking buffer and added to individual wells on a 96-well plate containing a gold-conjugated anti-frataxin monoclonal antibody (mAB) in each well. For whole blood samples, 100 µL of blood were lysed with 300 µL of Buffer A and 25 µL of the cleared-supernatant was added to individual wells of the 96-well plate. For measurement of frataxin protein in fractionated whole blood, 100 µL of each cell fraction was combined with 300 µL of Buffer A and 25 µL of the cleared-supernatant was added to individual wells of the 96-well plate. Tubes were incubated on ice for 30 minutes with intermittent vortexing and centrifuged at 20,000 g for 10 min at 4°C to remove insoluble material. Samples were incubated for 5 minutes at room temperature, allowing the gold-conjugated antibody to hydrate. After samples were mixed by gentle pipetting, a dipstick was added to each well and samples were allowed to wick up the membrane. Dipsticks were read using a Hamamatsu immunochromato reader (Mitosciences dipstick reader, MS1000) and the amount of frataxin quantified.

Using the frataxin ELISA (Abcam), 15 µg of protein derived from PBMCs in 200 µL of ELISA incubation solution was added to individual wells of the microplate. For whole blood, 80 µL of blood was lysed with 240 µL ELISA lysis buffer then the lysate was cleared of insoluble material by centrifugation at 4°C. Patient and carrier samples were diluted 1∶2 with ELISA incubation solution, while control samples were diluted 1∶4 with ELISA incubation solution. 200 µL of each sample was added to individual wells of the microplate. Each sample was measured in duplicate. An rFXN standard curve was prepared and added to the microplate according to the manufacturer’s instructions.

### Frataxin RT-qPCR

The level of frataxin mRNA was measured using a TaqMan® Probe-based (Applied Biosystems, Carlsbad, CA), one-step real-time quantitative PCR (RT-qPCR) assay. Total RNA (50 ng) was combined with Brilliant II 1-step qRT-PCR master mix (Agilent Technologies, Santa Clara, CA), TaqMan Gene Expression Assay for frataxin (Hs00175940_m1), TaqMan Gene Expression Assay for GAPDH (Applied Biosystems), and reverse transcriptase (Agilent Technologies) in a 25 µL reaction volume. Assays were performed in duplicate using an Mx3005 or Mx3000 instrument and analyzed using MxPro Software (Agilent Technologies). The amount of frataxin mRNA in each sample was determined as the relative quantity to the calibrator where the calibrator sample was assigned an arbitrary quantity of 1. For baseline frataxin level measurements, RNA from control donors was the calibrator and for frataxin level measurements in HDACi-treated PBMCs, RNA from a DMSO-treated sample was the calibrator. Each plate contained a No Template Control, and a No Reverse Transcriptase Control was run for each sample. The relative quantity of frataxin mRNA was normalized to RNA input and GAPDH when baseline levels of frataxin were measured or to cell number and RNA input when the levels of frataxin were measured in PBMCs following treatment with HDAC inhibitor.

### Statistical Analysis

For analysis of frataxin protein and mRNA stability, frataxin protein and/or mRNA was measured from whole blood in two different cohorts at regular intervals over the course of four weeks (Cohort 1; n = 31) and 15 weeks (Cohort 2; n = 10). Differences in frataxin levels between visits were analyzed using one-way ANOVA for repeated measures with the Greenhouse-Geiser correction for sphericity. Last observation carried forward was used in the event of a missed clinical visit.

A further measure of frataxin protein stability, expressed as intra-individual variability over the duration of the study, was determined by measuring the coefficient of variation (CV) and comparing the value to previously reported ranges for the assay [19]. All CV values were defined as the ratio of the standard deviation/mean over the duration of the study.

In order to investigate the ability of frataxin level to predict disease status and progression, multivariate linear regression models were constructed using neurological status and measured by FARS exam or change in FARS exam as the dependent variables, and age, sex and frataxin level as independent variables as described previously [22], [24]. In this analysis, to capture an accurate, potentially linear rate of progression, we excluded patients with less than two years of FARS measurements (with fewer than three total visits) to reduce variation in disease progression and patients with FARS scores greater than 100 to eliminate a possible ceiling effect.

All data analyses were performed using Stata SE 11 software, Microsoft Office Excel 2007, and GraphPad Prism 5.0. A p-value of <0.05 was considered statistically significant.

## Results and Discussion

### Relation of Clinical Information to Frataxin Levels

Clinical and demographic information for the FRDA patients in Cohort 1 in this study is summarized in Table 1. Patients in Cohort 1 (n = 31) were observed systematically over four weeks to examine short-term reproducibility and stability of frataxin measurements and to determine the extent to which demographic information correlates with disease severity or frataxin protein level. A strong inverse hyperbolic correlation (R^2^ = 0.794, p<0.001) between age of disease onset and GAA repeat length on the shorter allele (GAA_1_) was observed, in agreement with previous studies [19], [25]–[27]. An inverse correlation was observed between GAA_1_ repeat length and frataxin protein level measured in whole blood from patients in Cohort 1 (R^2^ = 0.58, p<0.001). Although GAA repeat length highly correlated with age of onset and the level of frataxin protein, there was only a moderate direct correlation (R^2^ = 0.38, p<0.001) between age of onset and level of frataxin protein. The duration of disease did not correlate with frataxin protein levels, indicating that this cohort contains patients at various stages of disease progression.

**Table 1:** Summary of Clinical and Demographic Values from FRDA Patients at the Children’s Hospital of Philadelphia (Cohort 1).

	GAA_1_	GAA_2_	Age	Age of Onset	Age of Diagnosis*	Duration of Disease	Frataxin, % of Control
**Min**	126	359	18	5	8	2	5.1
**Max**	901	1450	58	41	46	30	91.2
**Median**	549	848	29	13	17	11	38.4
**Mean**	534.9	863.1	31.8	15.7	18.4	11.2	38.5

Demographic information for subjects at Children’s Hospital of Philadelphia study (n = 31);

* Denotes n = 24 for those particular demographic data; Sex was evenly distributed (13 female/13 male).

### Frataxin Protein and mRNA Expression is Stable Over Time

**Figure 1:**
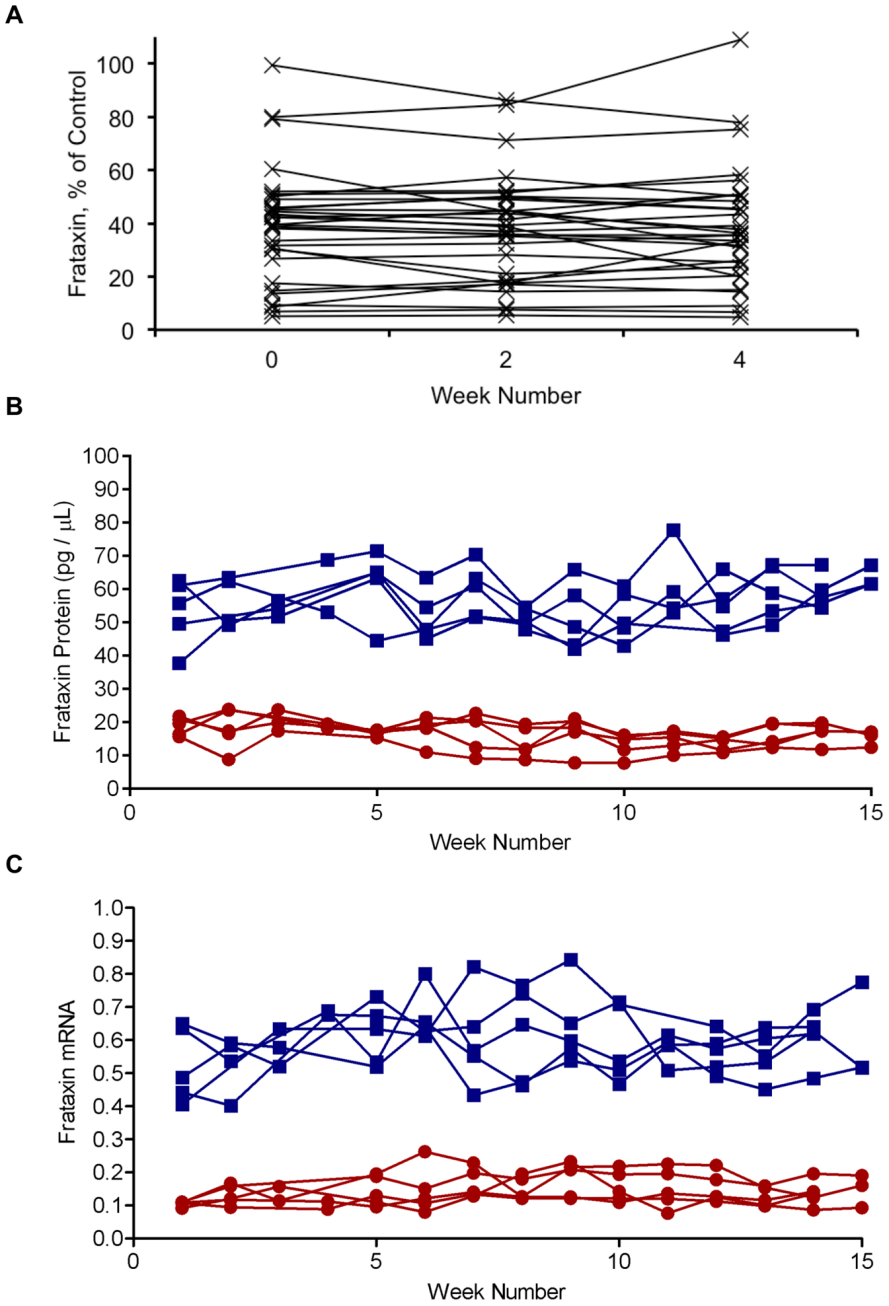
Frataxin protein and mRNA level is stable from day-to-day in whole blood. (A) Frataxin protein levels measured by dipstick assay in FRDA patient subjects over the course of 4 weeks (Cohort 1; n = 31). (B) Frataxin protein levels measured by dipstick assay in whole blood collected from 5 FRDA patients (red) and 5 related carriers (blue) weekly for 15 weeks (Cohort 2). (C) Frataxin mRNA levels measured by RT-qPCR in blood collected in PAXgene tubes from Cohort 2. Frataxin mRNA level is expressed relative to control donor levels and is normalized to the endogenous control gene *GAPDH*.

Frataxin protein level measured in whole blood from patients in Cohort 1 remained stable over the course of three clinic visits conducted over a period of 4 weeks (Figure 1A). Based on one-way ANOVA for repeated measures, there were no significant differences in frataxin levels between any of the clinical visits in Cohort 1 (p = 0.352). The intra- and inter-assay variability was within the previously reported range for this assay [19]. These data suggest that frataxin protein level is stable over a broad range of disease severity and do not demonstrate appreciable day-to-day variability.

To examine longer-term stability of frataxin protein and mRNA, measurements were made in whole blood collected from a second cohort (Cohort 2) on a weekly basis for 15 weeks. One-way ANOVA for repeated measures analysis revealed no statistically significant changes in frataxin protein (p = 0.068) or mRNA (p = 0.148) levels over the course of the study. In cohort 2, little variability was observed in frataxin protein level over the period of sample collection (Figure 1B). The average CV over the duration of the study was 15.6%, within the reported variability of the assay. While frataxin mRNA levels in whole blood collected in PAXgene tubes were also stable over this duration (Figure 1C), the variability was moderately higher than that observed for frataxin protein (15.6% CV compared to 18.0% CV for protein and mRNA, respectively).

### The Erythrocyte Fraction is the Major Contributor to Whole Blood Frataxin Protein

The level of frataxin protein in whole blood represents the weighted average of the different components of blood (PBMCs, platelets, erythrocytes, etc.). However, due to differences in the number of mitochondria in each cell type, the numbers of each cell type in blood, and potentially different expression levels between cell types, certain cells may contribute more to the overall level of frataxin protein than others. In addition, not all cell types in blood contain a nucleus; therefore, not all cells in a whole blood sample can directly increase the level of frataxin in response to HDACi therapy. This is a major consideration when using peripheral blood to monitor biologic activity for any therapeutic aimed at increasing frataxin.

Therefore, we sought to determine the contribution of each cell compartment to the total level of frataxin protein in blood using separation of cell types by differential centrifugation. The majority of the frataxin protein in whole blood was found in the erythrocyte fraction at 78.6 ng/mL or 85% of the whole blood frataxin signal. Platelets and PBMCs had lower frataxin levels at 7.0 ng/mL (8.5%) and 5.5 ng/mL (6.7%), respectively. Last, the granulocyte fraction had the least frataxin protein at 0.3 ng/mL or 0.4%. However, separation of blood using Ficoll is not an efficient means of granulocyte isolation, and thus the actual contribution of granulocytes might be underestimated.

Since the majority of frataxin measured in whole blood is found in erythrocytes, we explored measurements of frataxin in PBMCs and cheek swabs, as frataxin in erythrocytes is unlikely to change rapidly with therapeutics designed to increase transcription of the *FXN* gene, like HDAC inhibitors.

### Baseline Frataxin Protein and mRNA are Equivalent in Whole Blood, PBMCs, and Cheek Swabs

**Figure 2:**
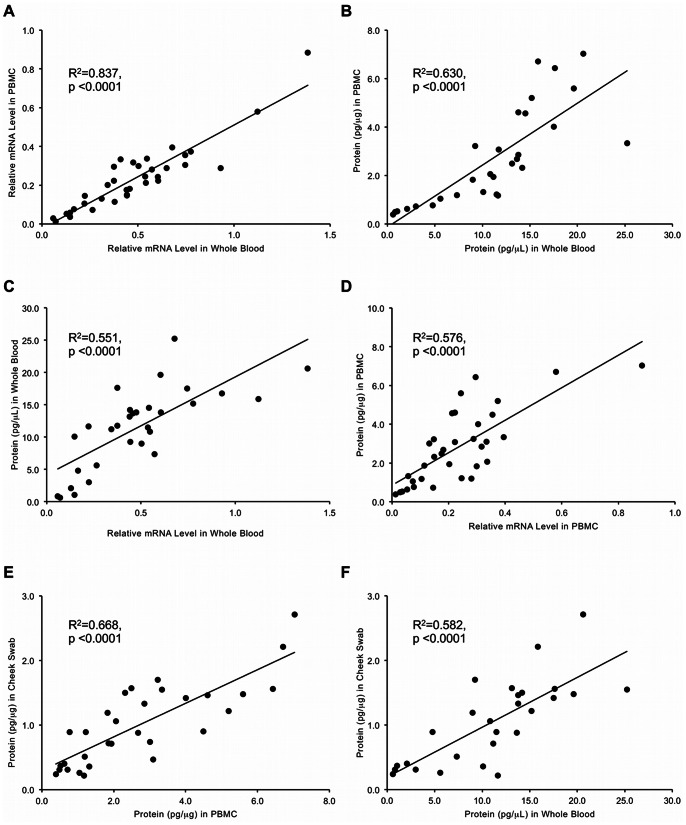
Frataxin protein and mRNA levels strongly correlated when measured in different cellular compartments (Cohort 3). (A) Frataxin mRNA in PBMCs and whole blood (R^2^ = 0.84, p<0.0001). (B) Frataxin protein in PBMCs and whole blood (R^2^ = 0.63, p<0.0001). (C) Frataxin mRNA and protein in whole blood (R^2^ = 0.55, p<0.0001). (D) Frataxin mRNA and protein in PBMCs (R^2^ = 0.58, p<0.0001). (E) Frataxin protein in PBMCs and cheek swabs (R^2^ = 0.67, p<0.0001). (F) Frataxin protein in whole blood and cheek swabs (R^2^ = 0.58, p<0.0001). Frataxin mRNA level in PBMCs and whole blood is expressed relative to control donor levels and is normalized to the endogenous control gene *GAPDH*.

In order to assess the relevance of measuring frataxin in one sample type over another, we compared frataxin mRNA and protein levels in whole blood, isolated PBMCs, and cheek swabs from a panel of FRDA patients, carriers and controls (Cohort 3). Baseline frataxin mRNA in PBMCs highly correlated with frataxin mRNA in whole blood (R^2^ = 0.84, p<0.0001) (Figure 2A). Similarly, frataxin protein in PBMCs correlated with frataxin protein in whole blood (R^2^ = 0.63, p<0.0001) (Figure 2B). Additionally, frataxin mRNA and protein correlated in whole blood (R^2^ = 0.55, p<0.0001) (Figure 2C) and in PBMCs (R^2^ = 0.58, p<0.0001) (Figure 2D). Frataxin protein levels in cheek swabs correlated well with levels in PBMC samples (R^2^ = 0.67, p<0.0001) (Figure 2E) and whole blood (R^2^ = 0.58, p<0.0001) (Figure 2F). These results indicate that the frataxin mRNA or protein levels measured in one sample type predict levels in a different sample type. Additionally, these data support the accuracy of each measurement method and the use of buccal cells for frataxin protein biomarker analysis in therapeutic studies. We were not able to isolate quality total RNA from buccal cells and therefore could not assess frataxin mRNA levels in this sample type.

### HDACi-Induced Changes in Frataxin mRNA and Protein are Independent of GAA Repeat Length

**Figure 3:**
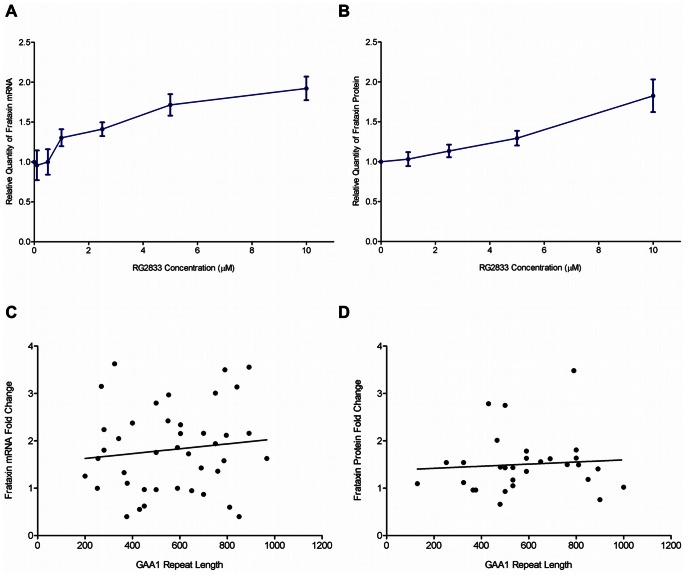
Effect of HDAC inhibitor RG2833 on frataxin level in PBMCs (Cohort 3). (A) The effect of RG2833 on frataxin mRNA level relative to vehicle control in PBMCs following 48 hour treatment (n = 49 at 1, 2.5, 5, and 10 µM; n = 2 at 0.1 and 0.5 µM). The relative quantity of frataxin mRNA in HDACi–treated PBMCs is normalized to cell number and RNA input. (B) The effect of RG2833 on FXN protein level relative to vehicle control in PBMCs following 72 hour treatment (n = 27). (C) The fold-change increase in FXN mRNA in PBMC in response to HDACi does not correlate with repeat length (GAA1, R^2^ = 0.015, p = 0.451). (D) The fold-change increase in FXN protein in response to HDACi does not correlate with repeat length (GAA1, R^2^ = 0.006, p = 0.686).

HDAC inhibitors such as RG2833 increase frataxin mRNA and protein in patient lymphocytes [13], [14] and in animal models of Friedreich’s ataxia [14]–[16]. We examined the variability in these measures across the patient population in Cohort 3 in order to better understand any potential variance seen in response to HDACi treatment in clinical trials. PBMCs were isolated from FRDA patient blood and cultured in the presence of 1–10 µM RG2833 or DMSO for 48 or 72 hours for frataxin mRNA or protein measurements, respectively. RG2833 treatment of patient derived PBMCs resulted in a dose-dependent increase in frataxin mRNA (n = 47; Figure 3A) expressed as an average fold change over vehicle control. With this method of PBMC culture and mRNA quantification, ≥1.3 fold change in mRNA is statistically significant. In this series of patient samples exposed to 10 µM RG2833, 83% (39 of 47) had increased frataxin mRNA after HDAC inhibitor treatment. We also observed similar drug-induced changes in frataxin protein levels in patient PBMC samples (n = 27; Figure 3B).

While it is useful to define the biochemical limit of detecting a response to drug treatment, it is important to use this *in vitro* response to estimate a potential therapeutic response during patient treatment. The levels of frataxin mRNA and protein observed in lymphocytes obtained from carriers are 1.5 to 2-fold higher than seen in patient lymphocytes (data not shown). If the RG2833 response rate *in vitro* is defined as the percent of patient PBMCs that show a 1.5 or 2-fold increase in frataxin in response to 10 µM RG2833 then the response rates are 72% and 50% of patients tested, respectively (Table 2). For protein, the response rates for the same fold change cutoffs are 58% and 23% response.

**Table 2:** Response Rate for RG2833 in PBMC Isolated From FRDA Patients in Cohort 3 and Treated *Ex Vivo*.

% Response to 10 µM RG2833		
	1.5-Fold Increase inFrataxin	2.0-Fold Increase inFrataxin
**Protein**	58	23
**mRNA**	72	50

Protein, n = 52; mRNA, n = 81.

In order to determine whether GAA_1_ repeat length could be used to predict the change in frataxin mRNA or protein in response to RG2833, GAA_1_ repeat length was plotted against frataxin response to RG2833 treatment (Figure 3C and 3D). There was no correlation between GAA_1_ repeat length and frataxin mRNA or protein response to 10 µM (R^2^ = 0.015 and R^2^ = 0.006, respectively). Similarly, baseline frataxin mRNA and response to RG2833 did not correlate (data not shown).

### Relation of Frataxin Levels to Disease Severity and Progression

Since frataxin levels are stable over time, reproducibly measurable in peripheral tissue, and responsive to potential therapeutic agents such as HDACi, we investigated whether frataxin levels would predict severity of disease as measured by FARS scores (Table 3). To do this we examined all patients in the Collaborative Clinical Research Network (CCRN) for FRDA patient registry, but excluded patients with less than two years of FARS measurements (with fewer than three total visits) to reduce variation in disease progression and patients with FARS scores greater than 100 to eliminate a possible ceiling effect [24]. In a linear regression model accounting for patient age and sex, frataxin levels in whole blood predicted baseline FARS scores (n = 94, p<0.001, R^2^ = 0.245). This finding was supported by the correlation between GAA_1_ and baseline FARS scores (p<0.001, R^2^ = 0.441), which suggests patients with longer GAA repeats and lower frataxin levels have a more severe neurological phenotype.

**Table 3:** Summary of Clinical Information and FRDA Disease Parameters by Frataxin Quartile.

Summary of Medians by Frataxin Quartile							
Quartile	N	Frataxin, % of Average Control	GAA_1_	Age of Onset	Age at Assessment	Baseline FARS	FARS, Change/Year (IQR)
**Q1**	23	11.2	857	7	20	64	2.9 (1.2–4.0)
**Q2**	24	22.0	600	11	19	59.5	2.1 (0.9–3.2)
**Q3**	24	31.0	480	16	27	50.2	2.0 (1.2–3.7)
**Q4**	23	48.7	325	19	49	48	1.6 (1.0–3.0)

Data collected from the CCRN for FRDA patient registry.

We then investigated whether frataxin levels would predict the rate of disease progression, as measured by rate of change in FARS scores. In previous work FARS scores were shown to increase at an average rate of 6.2±7.4 points per two-year period [22]. Age, but not GAA_1_ repeat length, was a significant predictor of change in FARS scores, indicating younger patients change faster for reasons not beyond their longer GAA repeat length. Here, we hypothesized that patients with lower frataxin levels would have slower disease progression as evidenced by a smaller change in FARS score per year. We found an overall rate of change in FARS scores of 2.3±2.5 points per year in patients with frataxin measurements (n = 94), similar to the previously reported rate [22]. Age was a significant predictor of disease progression; of FRDA patients with recorded frataxin measurements, adults progressed significantly slower (1.9±2.0 points per year) than children under the age of 18 (3.7±3.3 points per year, p = 0.003). On their own, frataxin levels did not predict change in FARS scores (p = 0.503); however, when using multivariate regression analysis to assess the effect of frataxin levels on rate of FARS change controlling for the effect of age and sex, the model was statistically significant (p = 0.002) with age as the main predictor of progression (p<0.001) and frataxin levels approaching significance (p = 0.096). Inclusion of frataxin level in the model increased the R^2^ value by 2.7% compared with a model not including frataxin values.

## General Discussion

In the present study we have defined the quantitative features of frataxin gene and protein measurement in relation to potential use as a marker of therapeutic response in FRDA. Methods to monitor the expression of the frataxin gene will thus be important supplements to clinical metrics, particularly in response to potential disease-modifying therapies such as HDAC inhibition. Previous reports have observed the inverse relationship of frataxin protein levels in blood with the size of the GAA repeat inserted in the frataxin gene and the age of onset of the disease [25]–[27]. In addition, clinical studies have investigated the effects of recombinant human erythropoietin on frataxin levels in PBMCs and lymphocytes utilizing ELISA-based methods for frataxin quantification, demonstrating that these assays are sensitive enough to detect changes in frataxin protein levels in response to pharmacological treatment; however, these studies were limited by their small sample sizes [20], [25], [28]–[32]. To-date, the practical uses of frataxin levels as a biomarker have not been fully characterized in studies involving a larger number of patient samples. This report documents for the first time the effects of an experimental therapeutic agent designed to change frataxin expression, HDAC inhibitor RG2833, on frataxin protein levels as measured by a lateral flow immunoassay.

For frataxin levels to be used as meaningful biomarkers in FRDA several features of the biochemical measure must be considered. The first critical aspect to using frataxin level to assess biological outcome of treatment with RG2833 or other disease modifying therapy is demonstrating the day-to-day stability of frataxin in the cells sampled in a clinical trial. Since a two-fold increase in frataxin is thought to be clinically relevant, it is important that the daily biological fluctuation of frataxin is low. Our data show that frataxin measurements as performed here meet this criterion.

A third requirement for use of frataxin as a biomarker in FRDA is that it must be responsive in a measurable way to interventions in a large proportion of subjects. Prior studies in animal models and PBMCs from patients had shown the ability to increase frataxin expression [14], [15], but here we show the first evaluation of drug response across a population of patient samples. We found that patients respond to HDACi, with 50% increasing frataxin mRNA 2-fold and 72% increasing frataxin mRNA 1.5-fold. Somewhat surprisingly, there was no correlation between GAA_1_ repeat length and frataxin fold-change in response to HDACi (Fig. 3C and 3D). These results suggest that GAA_1_ repeat length cannot be used to predict the degree of change in frataxin expression, and that the strength of pharmacological response is independent of disease severity. Finally for frataxin measurement to be a useful biomarker, it must predict disease severity and if possible change in disease severity. Here the data show that frataxin is a marker of disease severity in a cross sectional manner, and marginally predictive of ongoing change in neurological severity, though this analysis is made difficult by the interrelation between many variables (age, GAA repeat, length, age of onset, frataxin level) in a small sample size.

In summary, biochemical methods to quantify frataxin mRNA and protein levels have been developed and characterized across a population of FRDA patients. These observations suggest that frataxin levels can be used as a sensitive measure of treatment-induced expression changes. A preliminary evaluation of the *ex vivo* frataxin expression response rate across a population of FRDA patients suggest that ≥50% may respond to HDACi treatment with a measurable increase in frataxin. Additional characterization to correlate frataxin measures and clinical outcome will help define the relative significance of this biomarker in predicting clinical benefit from experimental drug treatments.
